# Molecular Mechanisms and Therapeutic Perspectives of Gut Microbiota, Autophagy, and Apoptosis in Cholangiocarcinoma Pathophysiology

**DOI:** 10.3390/ijms26209949

**Published:** 2025-10-13

**Authors:** Viviana A. Ruiz-Pozo, Santiago Cadena-Ullauri, Patricia Guevara-Ramírez, Rafael Tamayo-Trujillo, Elius Paz-Cruz, Alejandro Cabrera-Andrade, Ana Karina Zambrano

**Affiliations:** 1 Universidad UTE, Facultad de Ciencias de la Salud Eugenio Espejo, Centro de Investigación Genética y Genómica, Quito 170129, Ecuadorvictor.tamayo@ute.edu.ec (R.T.-T.);; 2 Grupo de Bio-Quimioinformática, Universidad de Las Américas, Quito 170125, Ecuador; raul.cabrera@udla.edu.ec; 3 Carrera de Enfermería, Facultad de Ciencias de la Salud, Universidad de Las Américas, Quito 170125, Ecuador

**Keywords:** microbiota, microbiome, cancer, autophagy, dysbiosis, apoptosis, cholangiocarcinoma

## Abstract

Cholangiocarcinoma (CCA) is an aggressive malignancy of the biliary tract with rising global incidence and limited treatment options. Its pathogenesis involves a complex interplay of genetic mutations, epigenetic dysregulation, inflammatory signaling, and environmental influences. Emerging evidence highlights the pivotal role of the gut–liver axis and microbiota dysbiosis in shaping biliary homeostasis and disease progression. Alterations in microbial composition disrupt apoptosis and autophagy, two key processes regulating cell survival and death, thereby contributing to tumorigenesis, metastasis, and therapy resistance. Specific taxa, including *Enterococcus*, *Escherichia coli*, *Pseudomonas*, *Bifidobacterium*, and *Bacillus*, demonstrate strain-dependent effects, acting either as tumor promoters through genotoxic metabolites and immune evasion or as potential tumor suppressors by inducing apoptosis and immune activation. These findings underscore the context-dependent roles of microbiota in CCA biology. Importantly, microbiota modulation offers novel therapeutic opportunities. Dietary interventions such as probiotics, prebiotics, and nutritional strategies, alongside innovative microbiome-targeted therapies, hold promise for restoring microbial balance, enhancing antitumor immunity, and improving patient outcomes. This review integrates current molecular and microbiological evidence to propose the gut microbiota as both a biomarker and a therapeutic target in CCA, opening avenues for precision medicine approaches in hepatobiliary oncology.

## 1. Introduction

Cholangiocarcinoma (CCA) is a heterogeneous group of malignant neoplasms originating from the cholangiocytes lining the biliary tree [1]. CCA is classified into two main types: intrahepatic cholangiocarcinoma (iCCA) and extrahepatic cholangiocarcinoma (eCCA). The extrahepatic type is divided into perihilar cholangiocarcinoma (also known as Klatskin tumor) and distal cholangiocarcinoma (dCCA). Each of these types has unique clinical, molecular, and prognostic characteristics [2,3].

CCA is the second most common primary hepatic malignancy, after hepatocellular carcinoma (HCC). Although it represents approximately 3% of all gastrointestinal cancers, its clinical impact is significant, as it accounts for approximately 15% of primary liver tumors [4]. CCA is generally diagnosed in advanced stages due to the absence of specific symptoms in the early phases, which contributes to its poor prognosis. In patients diagnosed in early stages, the five-year survival rate does not exceed 30% and decreases to 24% when regional lymph node involvement is present [5].

In recent decades, the incidence and mortality of CCA have progressively increased globally, with marked geographic differences. Asia has the highest rates, especially in northeastern Thailand (85 cases per 100,000 population), followed by South Korea [3,6]. High rates of cancer in these regions are likely influenced by endemic risk factors, particularly chronic infection with liver flukes associated with traditional dietary practices. These infections not only induce persistent biliary inflammation but also alter the intestinal and biliary microbiota, favoring a microenvironment that promotes carcinogenesis [7]. In North America, mortality rates from iCCA have shown average annual increases (AAPC) of 3.4% in men and up to 5.2% in women, in both the United States and Canada. In contrast, East Asia maintains high rates, but with a recent downward trend. For example, in South Korea and Hong Kong, declines in iCCA mortality have been reported, with AAPCs of −3.6% and −1.1% in men, respectively [6].

Several factors have been linked to the development of CCA, reflecting the complexity of its origin. These include chronic inflammatory diseases of the biliary tract, such as primary sclerosing cholangitis, choledochal cysts, and hepatolithiasis, as well as prolonged exposure to carcinogens like asbestos [5,8]. In certain regions of Southeast Asia, parasitic infections (by *Clonorchis sinensis* and *Opisthorchis viverrini*) acquired through consuming raw or undercooked fish have been linked to a higher incidence of CCA. Furthermore, metabolic conditions such as type 2 diabetes, obesity, and hepatic steatosis have also been recognized as risk factors. Despite these findings, many cases remain unexplained, underscoring the need for further research into the complex relationships between inflammation, metabolism, and environmental factors [8,9].

In this context, the study of the gut–liver axis and its interaction with the intestinal microbiota has gained relevance as a possible key modulator in the pathophysiology of CCA. The microbiota consists of microorganisms that inhabit various ecosystems within the human body, with the intestine being one of the most densely populated areas, hosting over a thousand bacterial species that interact symbiotically with the host [10,11]. This microbial ecosystem plays essential roles in digestion, metabolism, immune response, and epithelial barrier protection [12]. Given the anatomical and functional connection between the gut and liver, through portal circulation and enterohepatic recirculation of bile acids, it has been proposed that alterations in microbiota composition (dysbiosis) may promote chronic inflammation of the biliary epithelium, the translocation of microbial products, the disruption of biliary metabolism, and the activation of cellular pathways involved in tumorigenesis, such as apoptosis, autophagy, and epigenetic regulation [13,14,15]. This perspective presents new opportunities to understand the molecular mechanisms of CCA and develop innovative therapeutic strategies focused on microbiome modulation.

**Figure 1:**
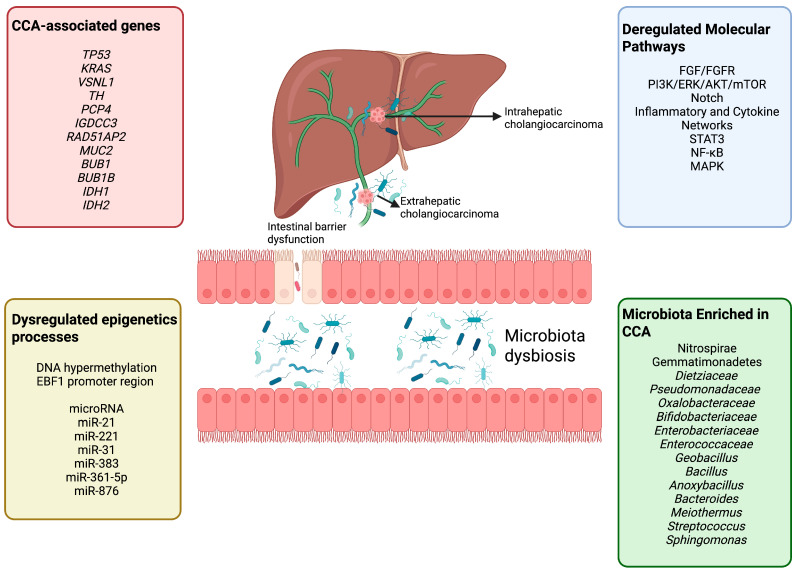
Molecular and microbial contributors to cholangiocarcinoma (CCA). Cholangiocarcinoma arises from a complex interplay of genetic alterations (e.g., *TP53*, *KRAS*, *IDH1/2*), dysregulated epigenetic processes (DNA hypermethylation, microRNAs), and aberrant signaling pathways (EGFR/FGFR, PI3K/ERK/Akt/mTOR, Notch, inflammatory networks). Furthermore, microbiota dysbiosis and intestinal barrier dysfunction contribute to biliary microbial enrichment, which could disrupt apoptosis, autophagy, bile acid metabolism, and immune regulation, potentially leading to intrahepatic and extrahepatic CCA development and progression. Created in https://BioRender.com.

This review aims to provide a detailed analysis of cholangiocarcinoma from a molecular and pathophysiology perspective, with special attention to the emerging role of the gut microbiota in its pathophysiology. It summarizes current evidence on how gut dysbiosis may influence inflammation, bile acid metabolism, and key cellular processes such as apoptosis and autophagy (Figure 1). Furthermore, it discusses microbiome-targeted strategies as potential tools to improve disease management.

## 2. Literature Search and Selection

This review was based on a comprehensive search on available databases, including Scopus, Google Scholar, PUBMED, and Web of Science up to August 2025. The keywords were: cholangiocarcinoma, biliary tract cancer, microbiota, gut–liver axis, autophagy, apoptosis, and signaling pathways. In cases of overlapping cohorts, the most comprehensive or recent study was cited. Priority was given to human studies specifically addressing CCA; however, mechanistic evidence was retrieved from other cancers, such as colorectal, gastric, hepatocellular, and from in vitro or animal models given the limited literature on cholangiocarcinoma.

## 3. Cholangiocarcinoma: Pathophysiology and Molecular Features

### Genetic and Epigenetic Alterations

CCA originates from a cellular modification process in different stages in which normal cholangiocytes accumulate genetic and epigenetic modifications that can accelerate the progression of CCA and worse prognosis [16]. Mutations in the tumor suppressor gene *TP53* are common across cancers and, in CCA, are associated with poor prognosis. In Asian populations, *TP53* mutations occur in approximately 38% of cases, a higher prevalence than in non-Asian cohorts [17].

Mutations in *KRAS*, an oncogene rather than a tumor suppressor, are present in 7–40% of CCA cases and correlate with worse survival in stages I–III [18]. Hotspot mutations are most often located in codons 12 and 13. In an analysis of 937 CCA patients, *KRAS* mutations were detected in 15.6% cases, with the most frequent allelic variants being G12D (37.0%), G12V (24.0%), and Q61H (8.2%) [19]. Mutations in *TP53* and *KRAS* occur at high frequency in CCA, and their co-occurrence in the same patient is linked to significantly reduced overall survival compared with alterations in only one of these genes [20].

Other genes also contribute to CCA pathogenesis. For example, research analyzed data from 36 patients with CCA tissue, and 9 normal tissues obtained from The Cancer Genome Atlas (TCGA) database, which revealed that the genes *VSNL1*, *TH*, *PCP4*, *IGDCC3*, *RAD51AP2*, *MUC2*, *BUB1*, and *BUB1B* are associated with tumor progression and with the prognosis of CCA [21].

Mutations in the *IDH1* and *IDH2* genes have been reported in 15–20% of CCA cases. particularly at IDH1 codons R132C and R132G [17]. Their frequency differs by subtype: 28% in intrahepatic CCA (iCCA) compared to 7% in extrahepatic CCA (eCCA) [17]. Their frequency differs by subtype: 28% in iCCA compared to 7% in eCCA [22]. Mutant IDH enzymes aberrantly convert isocitrate into the oncometabolite 2-hydroxyglutarate (2-HG), which accumulates to levels up to 100-fold higher than normal. This metabolite promotes carcinogenesis through epigenetic modifications, including DNA hypermethylation and altered histone modifications that block normal cellular differentiation [23].

Moreover, genetic differences exist between iCCA and eCCA. Small-duct iCCA is frequently characterized by mutations in *IDH1*, *IDH2*, and *FGFR2*, whereas large-duct iCCA and eCCA more commonly harbor mutations in *KRAS* and *TP53*, underscoring the importance of tumor subtype when interpreting genomic data [24,25].

Beyond genetic mutations, epigenetic mechanisms play a crucial role in CCA initiation and progression. Key epigenetic events include DNA hypermethylation and microRNA (miRNA) dysregulation.

DNA hypermethylation silences numerous tumor suppressor genes and cell-cycle regulators. This epigenetic silencing provides an alternative mechanism to genetic mutations for the inactivation of critical cellular controls [26]. For instance, *EBF1* is a transcription factor with tumor-suppressive functions. In CCA, hypermethylation of the EBF1 promoter region is significantly higher in tumors than in normal bile ducts, resulting in reduced mRNA expression. This epigenetic silencing decreases EBF1 protein production, thereby contributing to disease progression [27]. The p16INK4a gene undergoes hypermethylation in 14–50% of cases, leading to silencing of this key cell-cycle inhibitor. Similarly, p14ARF is epigenetically silenced by promoter methylation in approximately 38% of tumors. Collectively, such epigenetic inactivation of tumor suppressors and cell-cycle regulatory genes contributes to the initiation and progression of CCA [26].

Another important epigenetic mechanism in CCA involves microRNAs (miRNAs), small non-coding RNAs that regulate gene expression. miRNAs control key cellular processes, including apoptosis, migration, invasion, metastasis, and autophagy [28]. In CCA, several miRNAs are dysregulated, functioning either as oncogenes or tumor suppressors. For example, miR-21 is consistently overexpressed, promoting proliferation and metastasis by repressing tumor suppressor genes such as *PDCD4*, *PTPN14*, and *PTEN.* Elevated plasma levels of miR-21 correlate with larger tumor size and poor prognosis [28]. Similarly, miR-191 is upregulated in intrahepatic CCA, where it targets TET1, a tumor suppressor that normally limits progression [29]. Other oncogenic miRNAs, including miR-221, miR-31, miR-383, and miR-361-5p, contribute to tumor growth, migration, and chemoresistance [28].

In contrast, tumor-suppressive miRNAs are often downregulated in CCA. For example, miR-876 is reduced in CCA tissues and cell lines. Its target, BCL-XL, encodes an anti-apoptotic protein. Experimental overexpression of miR-876 suppresses BCL-XL, activates caspase-3/7, and triggers apoptosis, thereby reducing cell proliferation [30]. Thus, miRNA dysregulation represents a key molecular feature of CCA with potential diagnostic, prognostic, and therapeutic applications [31].

## 4. Critical Signaling Pathway Disruptions

The development and progression of CCA are driven by disruptions in key signaling pathways that regulate cell proliferation, survival, and invasion.

### 4.1. FGF/FGFR Pathway

The fibroblast growth factor/fibroblast growth factor receptor (FGF/FGFR) pathway regulates essential cellular processes, including survival, proliferation, differentiation, and metabolism [32]. This pathway comprises four receptors (FGFR1–4), which under physiological conditions are activated by FGFs. Ligand binding induces receptor phosphorylation and activates downstream cascades such as MAPK/ERK1/2, PI3K/Akt, and JAK–STAT [33,34].

Aberrant FGFR signaling contributes to CCA pathogenesis. Overexpression of FGF/FGFR has been associated with poor survival. Processes associated with the deregulation of this metabolic pathway include gain-of-function mutations, or gene fusion [35]. Among these, FGFR2 fusions—particularly FGFR2-BICC1—are the most common genetic alterations in intrahepatic CCA (iCCA), activating MAPK and PI3K/mTOR pathways. Interestingly, FGFR4 overexpression is observed in ~50% of cases. While generally linked to tumor progression, in iCCA it has been paradoxically associated with improved prognosis, suggesting its potential as a biomarker for a distinct molecular subtype [32].

### 4.2. PI3K/ERK/Akt/mTOR Pathway

The PI3K/Akt/mTOR pathway is activated by receptor tyrosine kinases (RTKs), which engage G proteins and RAS family members to stimulate PI3K activity. PI3K converts phosphatidylinositol-4,5-bisphosphate (PIP2) into phosphatidylinositol-3,4,5-trisphosphate (PIP3), which serves as a docking site for Akt. Subsequent phosphorylation by PDK1/2 kinases activates Akt, which in turn stimulates mTOR signaling by inhibiting the TSC1/2 suppressor complex [36].

mTOR functions within two multiprotein complexes. mTORC1 regulates protein synthesis, lipid and steroid biosynthesis, adaptation to hypoxia, epithelial–mesenchymal transition, and angiogenesis. mTORC2, activated primarily by growth factors, modulates intracellular kinases such as Akt and SGK1. This signaling axis is negatively regulated by PTEN, a tumor-suppressor phosphatase that dephosphorylates PIP3 back to PIP2, thereby restraining Akt activation and maintaining signaling balance [37].

In CCA, this pathway is frequently overactivated. An immunohistochemical study of 30 patients demonstrated overexpression of phosphorylated Akt and mTOR in tumor tissues compared with normal bile ducts, findings associated with poor prognosis. Mechanisms underlying this dysregulation include activating mutations or amplifications in upstream RTKs, loss of PTEN function, and autocrine growth factor signaling [38]. Oncogenic alterations within the RAS/RAF/MEK/ERK cascade and FGFR fusions converge on the PI3K/Akt/mTOR axis, resulting in sustained pathway activation that promotes uncontrolled proliferation, tumor progression, and metastatic dissemination [39].

### 4.3. Notch Pathway

The Notch signaling cascade is essential for biliary development and cholangiocyte differentiation. It consists of four receptors (Notch1–4) and five ligands (JAG1, JAG2, DLL1, DLL3, and DLL4), all of which are transmembrane proteins [40]. This ligand-receptor binding triggers a series of proteolytic cleavages that culminate in the release of the Notch intracellular domain (NICD). The NICD is transported to the nucleus, where it is associated with CSL transcription factors and coactivators such as Mastermind-like (MAML) proteins. This complex activates the transcription of target genes, including members of the Hes and Hey families, which regulate proliferation, apoptosis or differentiation [41,42,43].

In cholangiocarcinoma, aberrant Notch signaling has been strongly implicated in tumor initiation and progression. In intrahepatic CCA (iCCA), activation of this pathway promotes the conversion of mature hepatocytes into malignant cholangiocytes [40]. Overexpression of NOTCH1 together with its ligand JAG1 has been reported in human CCA cell lines, supporting a role in oncogenic signaling. Similarly, elevated expression of NOTCH3 has been associated with iCCA development through non-canonical activation of the PI3K/Akt pathway [44].

Notably, these molecular pathways do not act in isolation and can be influenced by external factors, including microbiota [45]. For instance, research has shown that microbial metabolites such as lipopolysaccharides (LPS), and short chain fatty acids (SCFAs) can alter these pathways in various types of cells, including hepatobiliary cells [46]. Thus, dysbiosis may influence cancer cell behaviors including apoptosis, survival, and immune evasion, which may be already driven by genetic alterations [46,47].

### 4.4. Inflammatory and Cytokine Networks

Chronic inflammation generates oxidative stress and DNA damage, promoting the proliferation of cholangiocytes and, over time, increasing the risk of malignant transformation. Proinflammatory cytokines, such as interleukin (IL)-6, tumor necrosis factor alpha (TNF-α), and interferon gamma (IFN-γ), activate signaling pathways such as STAT3, NF-κB, and MAPK, all of which contribute to survival, proliferation, and resistance to apoptosis in cholangiocytes [48].

Signal transducer and activator of transcription 3 (STAT3) is a transcription factor that mediates cytokine signaling. It is primarily activated by phosphorylation at Tyr705, which promotes dimerization and nuclear translocation, while phosphorylation at Ser727 enhances its transcriptional activity [49]. In the nucleus, STAT3 homodimers or heterodimers bind DNA and induce the expression of target genes. Under physiological conditions, STAT3 activation is transient; however, in CCA, it is constitutively activated, driving tumor progression through sustained transcription of oncogenic and survival-related genes [50].

Multiple pro-inflammatory and oncogenic pathways contribute to aberrant STAT3 activation in CCA. IL-6, together with its soluble receptor, activates GP130 and the JAK/STAT3 cascade, leading to inflammatory cytokine expression, apoptosis resistance, and tumor growth. Similarly, IL-10, secreted by immune cells and M2 macrophages, induces STAT3 activation in regulatory T cells and tumor cells, promoting migration, invasion, and epithelial–mesenchymal transition (EMT). In addition, EGFR signaling phosphorylates STAT3 at Tyr705, enhancing transcriptional activity and promoting proliferation, angiogenesis, and cell survival [49,51].

Dysbiosis and the metabolites generated by these bacterial species can further influence inflammatory pathways [52,53]. For instance, studies have found that LPS has been detected in a variety of tissues from cancer patients and can interact with inflammatory pathways and promote invasion and cell migration in cholangiocarcinoma patients, affecting their overall survival [54].

### 4.5. Gut-Liver Axis and the Microbiota in Hepatobiliary Disease

The gut–liver axis refers to the bidirectional communication between the intestine and the liver through several pathways, the most important via is the portal circulation [55,56]. This system exposes the liver to a wide range of substances derived from the gastrointestinal tract including nutrients, enterobacteria-related substances, cytokines and others [56]. Liver acts as a biological barrier, filtering harmful factors from entering the body [56]. Lifestyle factors such as high-fat diets, alcohol consumption and physical inactivity can disrupt the intestinal microbiota, leading to an intestinal barrier dysfunction [55]. Communication is reciprocal, as the liver directly influences the gut through the biliary system. Hepatocytes synthesize and secrete bile, a fluid rich in immunoglobulin A (IgA), bicarbonate antimicrobial molecules and bile acids. Once secreted, bile acids can enter the gut to aid in lipid digestion and are transformed by gut bacteria dehydroxylate into secondary bile acids. These metabolites are reabsorbed into the liver and activate intestinal receptors such as farnesoid X receptor (FXR) and Takeda G protein-coupled receptor 5 (TGR5), regulating lipid and glucose metabolism [55,57]. At the same time, the portal circulation delivers nutrient, lipids, microbial products such as LPS and short-chain fatty acids from the gut to the liver [55].

Under physiological conditions, multiple gut barrier layers prevent bacterial translocation to the liver. These include intestinal epithelium, mucus layer, lamina propria which contains plasma cells that produce IgA that neutralize pathogens and gut-vascular barrier [55]. However, in disease states, these defenses can be compromised. Barrier disruptions allow bacteria and their products to translocate into mesenteric lymph nodes, portal, and systemic circulation [58]. In the liver, portal blood flows toward the central vein via sinusoids, where Kupffer cells and sinusoidal endothelial cells serve as a second firewall by clearing translocate microbes and releasing chemokines to recruit immune cells [55]. Persistent microbial translocation, however, overwhelms these defenses and promotes chronic hepatic inflammation along the gut–liver axis.

There is growing evidence that dysbiosis plays a role in hepatobiliary diseases. In non-alcoholic fatty liver disease (NAFLD), microbiome alterations vary according to disease stage. For example, a clinical study reported that patients with NAFLD-cirrhosis had increased *Streptococcus* and *Gallibacterium*, while patients with NAFLD without advance fibrosis had higher levels of *Streptococcus*, *Bacillus* and *Lactococcus* [59]. In a murine study, Zhang et al. demonstrated that *Bifidobacterium* and *Bacteroides* decreased, while *Mucispirillum*, *Desulfovibrio*, *Anaerotruncus* and *Desulfovibrionaceae* increased [60]. Another study in patients with nonalcoholic steatohepatitis (NASH) found reduced abundances of *Faecalibacterium* and *Anaerosporobacter*, accompanied by increases in *Parabacteroides* and *Allisonella* [61].

In cirrhosis, dysbiosis is strongly associated with disease progression and prognosis. One study showed that patients with dysbiosis had higher mortality, characterized by increased *Enterobacteriaceae*, and *Lactobacillaceae*, alongside decreased *Ruminococcus* and *Lachnospiraceae* [58]. Another study comparing survivors and non-survivors with cirrhosis found that deceased patients had higher abundance of *Propionibacteriaceae* and *Halomonadaceae* and lower levels of *Lachnospiraceae* and *Veillonellaceae* [62].

Emerging data also implicate the microbiome in CCA. A comparative study of intrahepatic cholangiocarcinoma and hepatocellular carcinoma gut microbiota showed that intrahepatic cholangiocarcinoma was associated with increased *Veillonellaceae*, *Lactobacillales*, *Actinomycetaceae*, *Streptococcaceae*, and *Neisseriaceae* (*V. atypica* and *V. parvula)*, and reduced *Lachnospiraceae* and *Eubacteriaceae*, while hepatocellular carcinoma samples had higher levels of *Blautia* [63]. Oral microbiome analyses in CCA patients revealed increases in *Streptococcus*, *Veillonella*, *Haemophilus*, *Leptotrichia*, *Granulicatella*, *Capnocytophaga* and *Alloprevotella* and reductions in *Actinomyces* [64]. Bile microbiota characterization identified potential biomarkers in perihilar cholangiocarcinoma: *Pseudomonas*, *Sphingomonas*, and *Halomonas* [65]. *Bacteroides*, *Geobacillus*, *Meiothermus*, and *Anoxybacillus* were elevated in bile microbiota in extrahepatic cholangiocarcinoma compared with controls [66]. In distal cholangiocarcinoma, studies have shown increased bacteria in bile, including *Gemmatimonas*, *Nitrospira*, *Chloroflexus*, and *Planctomyces*, compared to controls [67]. Moreover, bile duct tissue from CCA patients showed increased abundance of *Dietziaceae*, *Pseudomonadaceae* and *Oxalobacteraceae*, whereas gastric mucosa tissue was dominated by *Moraxellaceae* and exhibited lower abundances of *Burkholderiaceae* [68].

In summary, multiple hepatobiliary disorders are characterized by gut dysbiosis, suggesting that microbial imbalance and translocation along the gut–liver axis play central roles in disease pathogenesis (Table 1).

## 5. Modulation of Autophagy and Apoptosis by Gut Microbiota

**Figure 2:**
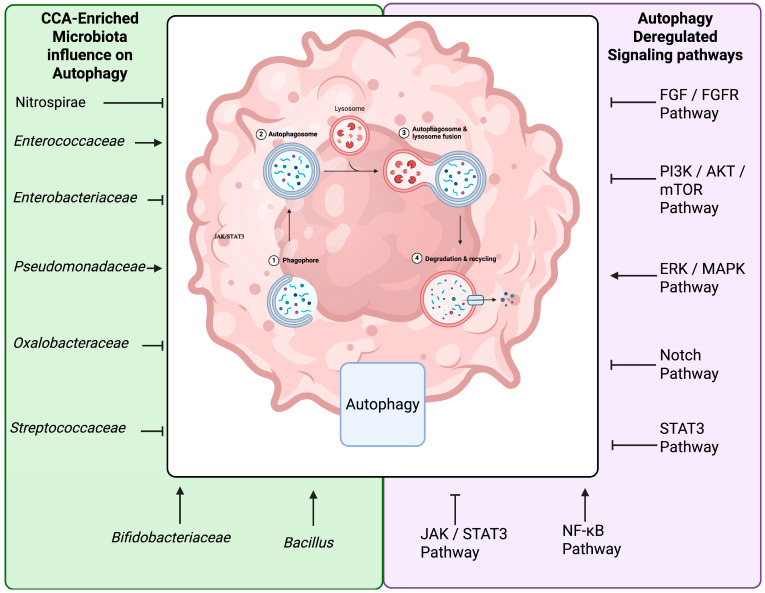
Cholangiocarcinoma-enriched microbiota and signaling pathways influencing autophagy in cholangiocarcinoma. The schematic illustrates the four main stages of autophagy in eukaryotic cells: (i) initiation and formation of the phagophore, (ii) elongation and closure into the autophagosome, (iii) fusion of autophagosomes with lysosomes, and (iv) degradation and recycling of sequestered material by lysosomal hydrolases. Microbial taxa enriched in cholangiocarcinoma, such as *Nitrospirae*, *Enterobacteriaceae*, *Oxalobacteraceae*, and *Streptococcaceae*, may inhibit different steps of autophagy, while commensal taxa such as *Enterococcaceae*, *Pseudomonadaceae*, *Bifidobacteriaceae* and *Bacillus* may promote autophagy. Autophagy in CCA plays a dual role, functioning either as a promoter or suppressor of carcinogenesis. As a tumor-suppressive mechanism, it reduces genomic instability and facilitates the clearance of damaged organelles [73]. Conversely, autophagy can promote tumor growth by sustaining cancer cell survival under stress conditions and by contributing to the development of drug resistance [74,75]. Deregulated intracellular signaling pathways, including EGFR/FGFR, PI3K/Akt/mTOR, ERK/MAPK, Notch, JAK/STAT, and NF-κB, further disrupt autophagy regulation and contribute to tumor progression. Created in https://BioRender.com.

Autophagy is an evolutionarily conserved intracellular pathway that is activated in response to a variety of environmental and cellular stimuli [69,70,71]. Evidence has shown that autophagy also participates in a wide range of cellular processes, including apoptosis, and cancer biology [69,70]. In the context of CCA, impaired or dysregulated autophagy has been strongly associated with changes in cellular behaviors of cholangiocarcinoma, affecting processes such as proliferation, metastasis, apoptosis, and drug response, by interacting with molecular signaling pathways (Figure 2) [69,70,71,72].

**Figure 3:**
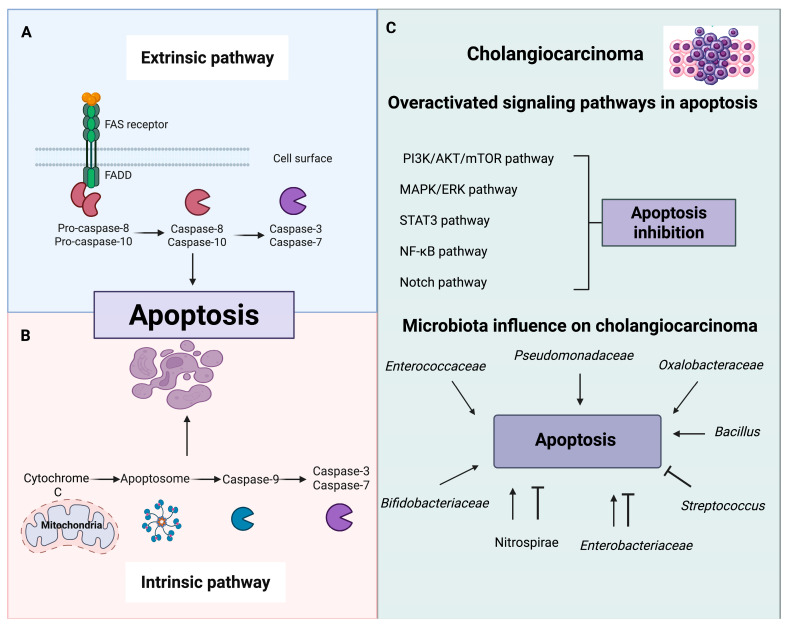
Extrinsic and intrinsic apoptotic pathways and their influence on cholangiocarcinoma (CCA). Apoptosis proceeds through two major pathways: (**A**) the extrinsic pathway, initiated by Fas receptor activation, recruitment of FADD, and cleavage of pro-caspases-8/10, which in turn activate the executioner caspases-3 and 7; and (**B**) the intrinsic pathway, triggered by mitochondrial release of cytochrome c, leading to apoptosome assembly, caspase-9 activation, and subsequent activation of caspases-3 and 7. Both pathways converge on executioner caspases to induce programmed cell death. (**C**) In CCA multiple mechanisms disrupt the apoptosis. Key signaling cascades commonly dysregulated in cholangiocarcinoma (PI3K/Akt/mTOR, MAPK/ERK, STAT3, NF-κB, and Notch), which inhibit apoptosis and enhance tumor survival. Loss of the tumor suppressor *TP53* is a major mechanism underlying resistance to apoptosis, whereas oncogenic *KRAS* activation promotes uncontrolled cell proliferation, inhibits apoptotic signaling, and reduces sensitivity to anticancer therapies, ultimately contributing to poor prognosis in CCA [131,132]. Beyond genetic alterations in the biliary and gut microbiota can either suppress or promote apoptosis depending on bacterial metabolites and virulence factors, thereby contributing to CCA progression or, conversely, exerting anticancer effects. Created in https://BioRender.com.

Apoptosis, an ordered form of programmed cell death, has been extensively studied in relation to cancer [76,77,78]. Inhibition of apoptosis is documented across most cancer types [76,77,78]. Because apoptosis involves multiple signaling pathways, defects can promote malignant transformation, metastasis, and therapy resistance [76,77,78]. Therefore, current approaches are focusing on apoptosis modulation as a promising target for cancer treatment [77,78]. In this regard, bacteria have emerged as tools to modulate cell death pathways: outer membrane vesicles can serve as delivery systems for antitumor agents [79,80]. Furthermore, the enzymes, metabolites and toxins secreted by bacteria can interact with molecular pathways and lead to programmed cell death [81]. Thus, exploring bacteria-based strategies to promote tumor-selective apoptosis is promising (Figure 3).

Microbiota dysbiosis has been reported in several cancers [82]. In CCA, the biliary tract, considered sterile or nearly so, shows increased bacterial presence associated with disruption of the gut–liver axis, altered bile composition, and immune dysregulation, which together can contribute to cholangiopathies [83,84]. Regarding bile composition, evidence indicates that altered bile and specific microbes can inhibit apoptosis and increase predisposition to CCA [82].

In CCA, multiple taxonomic groups are enriched in bile and bile-duct tissues, indicating substantial biliary microbiome shifts. Phylum level: Nitrospirae and Gemmatimonadetes. Family level: *Dietziaceae, Pseudomonadaceae, Oxalobacteraceae, Bifidobacteriaceae, Enterobacteriaceae,* and *Enterococcaceae*. Genus level: *Geobacillus*, *Bacteroides*, *Meiothermus*, *Streptococcus*, *Sphingomonas*, *Bacillus*, and *Anoxybacillus*. Species level: *Enterococcus faecalis*, *Enterococcus faecium*, *Enterobacter cloacae*, and *Escherichia coli* [66,82,85].

To date, no studies have directly linked specific biliary microbiota to autophagy or apoptosis in CCA. However, various of these bacterial species, dysregulated in cholangiocarcinoma, have been shown to have autophagy and apoptosis modulatory properties in other types of cancer [69,82,83].

Within Nitrospirae (phylum), members participate in nitrification by oxidizing ammonia to nitrite and nitrite to nitrate [86,87]. In other tumor systems, nitrite/nitrate signaling has been shown to react with amines and amides to generate carcinogenic N-nitroso compounds [88]. Furthermore, nitrite/nitrate signaling could influence apoptosis, promoting or inhibiting it via oxidative stress and DNA damage or, conversely, by limiting lipid peroxidation and nitric oxide dynamics [89,90]. Similarly, nitrite can modulate autophagy through autophagy-related genes and mTOR-linked signaling [91]. Nitrospirae enrichment in CCA may suggest a tumor-promoting influence mediated by nitrosation chemistry and nitric oxide–related modulation of apoptosis and autophagy; however, this relationship remains hypothetical as direct evidence in CCA is lacking.

The family *Enterococcaceae*, particularly *Enterococcus faecalis*, and *Enterococcus faecium* have been reported to be increased in CCA [66,82,85]. *E. faecalis* appears in probiotic formulations with beneficial effects; however, certain strains can resist intra-phagosomal killing, surviving in low pH environments [92]. In non-CCA settings, research has shown that *E. faecalis* can activate autophagy in macrophages by inhibiting the PI3K/Akt/mTOR signaling pathway [92]. Additionally, *E. faecalis* can generate reactive oxygen species, which can cause DNA damage and lead to carcinogenesis [93,94].

Furthermore, *E. faecium* secretes enterocins, which have been correlated with antimicrobial activity and increased cellular apoptosis through suppression of anti-apoptotic proteins such as BCL-2 [95]. This species can promote apoptosis in several cancer cell types, including HT-29 colorectal adenocarcinoma, ovarian cancer cells, and AGS gastric adenocarcinoma [96,97]. The impact of *Enterococcaceae* in CCA remains uncertain; observations from other tumor systems suggest that strain-specific effects on autophagy or apoptosis may occur. Further work is required to clarify their role in the biliary niche. Thus, the enrichment of *Enterococcaceae* in CCA may suggest a context-dependent influence, with some strains potentially promoting carcinogenesis via reactive oxygen species (ROS) and autophagy modulation, while others may exert anticancer effects by inducing apoptosis.

*Enterobacteriaceae*, including *Enterobacter cloacae* and *Escherichia coli*, have been reported as enriched in CCA patients [66,82,85]. *E. cloacae* have been shown in non-CCA models to induce apoptosis across multiple cancer cell types, including intestinal epithelial, osteosarcoma, and cervical cancer cells. Furthermore, *E. cloacae*-derived compounds can trigger cytotoxicity against human myeloid leukemia via PARP degradation, caspase-9/3 activation, increased Bax/Bcl-2 ratio, and p53 upregulation [98,99,100,101].

For *Eschericia coli*, part of this family, increased abundance in CCA has been described; however, the mechanistic evidence on apoptosis and autophagy modulation derives from other types of cancer and in vitro studies [66,82,85,102,103,104,105]. For autophagy, the HlyF protein promotes overproduction of outer membrane vesicles that inhibit autophagosome–lysosome fusion, blocking autophagy [102]. In vitro studies have shown that *E. coli* can reprogram the tumor microenvironment by enhancing migration via epithelial–mesenchymal transition [103]. Colibactin, a genotoxic metabolite, promotes invasive colonic tumors and inhibits autophagy in wild-type mice [104,105]. Conversely, extracellular vesicles from some *E. coli* strains induce oxidative stress and mitochondrial autophagy, limiting cell growth in adenocarcinoma models [106].

Regarding apoptosis, *E. coli* OmpA protein can downregulate Bak, Bax, and p53, disrupting apoptotic pathways and potentially facilitating metastasis in colorectal cancer patients [107]. In contrast, certain pks+ strains may promote apoptosis and cell-cycle arrest in colon cancer cell lines [108]. Together, these data suggest that strain-specific virulence factors can either promote or suppress apoptosis and autophagy; whether these mechanisms apply to CCA remains to be determined.

*Pseudomonadaceae* is also enriched in CCA patients [66,82,85]. Research has shown, in non-CCA settings, that various species of this family interact with autophagy and apoptosis pathways. For instance, *Pseudomonas aeruginosa* type III effector ExoS can induce apoptosis through Bax/Bim upregulation, mitochondrial membrane disruption, and caspase-9/3 activation [109]. Mouse-derived cancer cell models infected with *P. aeruginosa* show increased autophagy and apoptosis through MAPK signaling [110]. Moreover, *Pseudomonas aeruginosa mannose-sensitive-hemagglutinin* has shown promise against various types of cancer, including breast and lung cancer by promoting cell-cycle arrest and activating caspase-9 and caspase-3 [111]. However, *Pseudomonas* species have shown the ability to modulate apoptosis and autophagy in other tumor systems, direct mechanistic data in CCA is absent, highlighting the need of further CCA studies.

*Oxalobacteraceae* are also reported to be increased in proportion in CCA patients [66,82,85]. Violacein, a pigment produced by several *Oxalobacteraceae* members, has been linked to enhanced apoptosis and reduced autophagy in diverse cancer cell lines [112,113,114]. These effects include caspase-3 activation and persistence even in the presence of oncogenic BRAF or RAS mutations, with signaling through RAS–RAF–MEK–ERK and Akt pathways [112,113,114]. Although *Oxalobacteraceae* enrichment has been observed in some CCA datasets, its functional significance remains unclear. Evidence from other models suggests that violacein-producing strains might exert anticancer effects; however, no direct CCA studies have determined these mechanisms within the biliary context.

For *Sphingomonas*, research has shown that this genus is increased in CCA [66,82,85]. Studies have reported that some *Sphingomonas* strains can induce apoptosis in human epithelial cells, as evidenced by chromatin condensation, sub-G1 DNA increase, and caspase-3 activation [115]. While their enrichment in CCA suggests possible relevance, the direct mechanisms in biliary tissue have not yet been characterized.

The proportion of the genus *Streptococcus*, belonging to the family *Streptococcaceae*, is also increased in CCA samples [66,82,85]. Various species of this genus have been associated with autophagy and apoptosis. For instance, group A *Streptococcus* can increase calpain activation, inhibiting autophagy and disrupting the capture of group A *Streptococcus* in autophagosomes [116]. Some strains of *Streptococcus* can reduce apoptosis by limiting the production of ROS and decreasing neutrophil activation [117]. On the other hand, some species belonging to this genus have been shown to promote autophagy in cancer cells, such as *Streptococcus anginosus* in oral squamous cell carcinoma [118]. These divergent effects underscore strain- and context-specificity, reinforcing the need for CCA-focused studies.

*Bifidobacteriaceae* enrichment has been detected in CCA patients [66,82,85]. Recent preclinical studies suggest that this family has the capacity to promote both autophagy and apoptosis [69,119,120]. For instance, in murine melanoma models, oral administration of *Bifidobacterium* suppressed tumor growth, with effects potentially comparable to antibody therapies [69]. Similarly, *Bifidobacterium* has been used as an adjuvant in hepatocellular carcinoma to inhibit tumor growth, increase IFN-γ secretion, and downregulate the JAK/STAT3 pathway [119]. In vitro, certain strains can induce autophagy in intestinal epithelial cells, suggesting mechanistic links relevant to barrier homeostasis [121]. While these effects have not yet been demonstrated in CCA, these findings are strain-specific and largely preclinical, and issues such as optimal dosing, safety in humans, and clinical efficacy remain undetermined.

For the genus *Bacillus*, also increased in CCA, multiple studies have examined mechanisms connecting the genus to autophagy and apoptosis [122,123]. Most supporting evidence comes from in vitro and animal studies, where *Bacillus*-derived lipopeptides and metabolites have induced apoptosis and autophagy in diverse cancer cell lines [124,125,126,127]. For instance, research has shown that *Bacillus amyloliquefaciens* may have immunomodulatory effects through autophagy induction in macrophages [122,123]. Although these mechanisms have not been tested in CCA or validated in clinical settings, it shows promise as a potential cancer management application; however, strain specificity, dose dependency, and safety considerations will be critical for translating these preclinical findings.

Throughout this section, evidence is predominantly preclinical; human data in CCA are limited, and translation will depend on strain specificity, dosing, and safety.

No information was found linking autophagy or apoptosis to Gemmatimonadetes (phylum), *Dietziaceae* (family), or the genera *Geobacillus*, *Anoxybacillus*, and *Meiothermus*.

Moreover, while several microbial taxa (e.g., *Nitrospirae*, *Enterococcus*, *Bifidobacterium*, *Bacillus*) are consistently enriched in CCA, the mechanistic evidence linking them to autophagy and apoptosis derives largely from other tumor systems. This distinction underscores a critical gap in knowledge and highlights the need for direct functional studies in CCA.

Furthermore, beyond individual taxa, microbial community-level changes have also been noted. Saab et al. (2021) reported an altered Firmicutes–Bacteroidetes ratio with increased Bacteroidetes, consistent with dysbiosis in CCA [66]. This supports the view that CCA involves both enrichment of particular bacteria and broader ecological imbalance. Notably, within Bacteroides, enterotoxigenic *Bacteroides fragilis* has been linked to carcinogenesis by promoting proliferation, inhibiting apoptosis, and increasing inflammation through E-cadherin disruption and IL-8 upregulation via STAT-mediated signaling [128,129,130].

In conclusion, CCA is accompanied by alterations in the biliary and gut microbiota, many of which exert context-specific effects on autophagy and apoptosis. These processes, central to tumor initiation and progression, can be either used by malignant cells or leveraged for therapeutic benefit. The enrichment of taxa such as *Enterococcus*, *Escherichia coli*, *Pseudomonas*, *Bifidobacterium*, and *Bacillus* illustrates the dual capacity of microbes to promote carcinogenesis or induce tumor-suppressive mechanisms, depending on their metabolic products, virulence factors, and interactions with host signaling pathways.

These findings highlight the gut microbiota as both a contributor to CCA pathogenesis and a promising therapeutic target. Importantly, dietary interventions aimed at modulating microbial communities, such as the use of prebiotics, probiotics, and tailored nutritional strategies, may hold potential to restore microbial balance, enhance molecular processes including apoptosis, and autophagy.

## 6. Challenges and Future Perspectives

Despite notable progress in understanding the relationship between microbiota and CCA, this field still faces multiple challenges that limit its clinical applicability. Current research has revealed consistent associations between intestinal or biliary dysbiosis and the presence or progression of CCA, employing tools such as 16S rRNA sequencing [85], patient-derived organoid models [133], and genetic profiling studies [83]. However, much of the evidence is based on exploration studies with small sample sizes, heterogeneous methodologies, and cross-sectional designs, making it difficult to establish strong causal relationships.

In the methodology, preanalytical and sampling factors represent a fundamental challenge for studying the biliary microbiome, as clinical interventions such as endoscopic access or stenting can greatly alter microbial composition. These devices may promote the growth of biofilm-forming bacteria, such as *Streptococcus* and *Fusobacterium* [134]. Additionally, antibiotics given during the perioperative period have varying effects and typically have a limited impact in cases of ischemic biliary injuries [135]. It is also important to consider the analyzed compartment, as significant differences have been reported between bile, ductal tissue, and feces. There is also a risk of contamination from duodenobiliary reflux, even when careful sampling techniques are used [136,137]. Technical aspects such as transport and storage conditions, DNA extraction methods, selection of the 16S hypervariable region, and bioinformatics decisions can increase heterogeneity between studies. Therefore, it is essential to standardize and systematically report these variables in future longitudinal, multicenter, prospective trials, to strengthen the validity of causal inferences and move towards more robust clinical applications [138,139,140].

Another significant gap in current research is the lack of longitudinal studies examining the dynamic effects of the microbiota throughout the clinical progression of CCA, as well as its potential influence on responses to conventional treatments. While pilot studies have suggested that certain microbial profiles could be associated with chemotherapy resistance, these findings require validation in larger and more diverse cohorts [141]. In particular, the environmental, dietary, and lifestyle factors that shape microbiome composition vary considerably across geographic regions and ethnic groups. Therefore, it is crucial to replicate these findings in multi-ethnic studies before applying them in clinical practice worldwide [142,143].

Furthermore, integrating functional metagenomics, transcriptomics, and metabolomics may help identify bioactive metabolites, inflammatory mediators, and microbiota-modulated immune pathways that are potentially implicated in biliary epithelial transformation and tumor progression. Moreover, the incorporation of patient-derived organoids and germ-free or gnotobiotic animal models represents a valuable tool for validating causal relationships and testing microbiome-modulating interventions in preclinical settings.

Finally, although the use of microbial signatures as noninvasive biomarkers for early diagnosis or prediction of therapeutic response is promising, it is still in the early stages of development [144]. Similarly, microbiome-targeted therapies such as probiotics, prebiotics, postbiotics, dietary interventions, and fecal microbiota transplantation still lack clear standards regarding their safety, efficacy, and clinical application in patients with CCA [145]. Combining these strategies with conventional treatments, targeted therapies, or immunotherapy could open up new possibilities within precision medicine, aimed at improving clinical outcomes. However, their implementation requires well-designed clinical trials and international collaborative efforts that consider the inclusion of underrepresented populations and recognize the geographic and cultural diversity in microbial composition and host-microbiota interactions.

## 7. Conclusions

Cholangiocarcinoma is a complex disease resulting from the interaction of genetic, epigenetic, immunological, and microbial factors. Additionally, dysregulation of autophagy and apoptosis, driven by gut and biliary microbiota alterations, contributes to tumor initiation, progression, and therapy resistance. The enrichment of taxa such as *Enterococcus*, *Escherichia coli*, *Pseudomonas*, *Bifidobacterium*, and *Bacillus* may reflect a dual role, acting as potential tumor promoters or suppressors depending on strain-specific and host-contextual factors; however, this duality remains to be demonstrated in CCA. These insights highlight the gut microbiota not only as a contributor to CCA pathogenesis but also as a promising therapeutic target. Notably, while microbial enrichment in CCA is documented, mechanistic effects on autophagy/apoptosis remain largely inferred from other tumor systems and require direct validation in CCA.

To strengthen future research, it is important to establish harmonized sampling and analysis protocols for bile, tissue, and feces. These protocols should not only consider technical aspects but also systematically integrate clinical metadata such as prior antibiotic use, stent placement, and ERCP approach. In parallel, the field will benefit from the development of biomarker-guided pilot studies exploring microbial or dietary interventions, incorporating markers of apoptosis and autophagy, such as cleaved caspase-3 or LC3/Beclin, in tissue or circulating samples. Integrating these strategies with therapies aimed at modulating the microbiota could help restore microbial balance and may favorably influence apoptosis and autophagy regulation.

## Figures and Tables

**Table 1:** Dysbiosis of Gut, Oral, and Biliary Microbiota in Liver Diseases and Cholangiocarcinoma.

Model	Liver Disease	Type of Microbiota and Sample	Dysbiosis at the Genus or Family Level Microbiome	Technique	Reference
Human	NAFLD	Gut microbiota Stool	NAFLD-cirrhosis: ↑ *Streptococcus* ↑ *Gallibacterium* NAFLD without advance fibrosis: ↑ *Streptococcus*, ↑ *Bacillus* ↑ *Lactococcus*	16S rRNA sequencing	[59]
Animal	NAFLD	Gut microbiota Stool	↓ *Bifidobacterium* ↓ *Bacteroides* ↑ *Mucispirillum* ↑ *Desulfovibrio* ↑ *Anaerotruncus* ↑ *Desulfovibrionaceae*	16S rRNA sequencing	[60]
Human	NASH	Gut microbiota Fecal	↑ *Faecalibacterium* ↑ *Anaerosporobacter* ↓ *Parabacteroides* ↓ *Allisonella*	16S ribosomal RNA pyrosequencing	[61]
Human	Cirrhosis survival vs. death	Gut microbiota Stool	↑ *Enterobacteriaceae*, ↑ *Lactobacillaceae* ↓ *Ruminococcus* ↓ *Lachnospiraceae*	16S ribosomal RNA sequencing	[58]
Human	Cirrhosis survival vs. death	Gut microbiota Stool	↑ *Propionibacteriaceae* ↑ *Halomonadaceae* ↓ *Lachnospiraceae* ↓ *Veillonellaceae*	Multi-tagged pyrosequencing techniques and ribosomal data (RDP10) taxa analysis	[62]
Human	Intrahepatic cholangiocarcinoma Hepatocellular carcinoma	Gut microbiota Stool	↑ *Veillonellaceae* ↑ *Lactobacillales* ↑ *Actinomycetaceae* ↑ *Streptococcaceae* ↑ *Neisseriaceae* ↓ *Lachnospiraceae*, ↓ *Eubacteriaceae* ↑ *Blautia*	Whole-genome metagenomic shotgun	[63]
Human	Cholangiocarcinoma	Oral microbiota Saliva	↑ *Streptococcus* ↑ *Veillonella* ↑ *Haemophilus* ↑ *Leptotrichia* ↑ *Granulicatella* ↑ *Capnocytophaga* ↑ *Alloprevotella* ↓ *Actinomyces*	16S rRNA sequencing	[64]
Human	Perihilar cholangiocarcinoma	Bile microbiota Bile sample	↑ *Pseudomonas*, ↑ *Sphingomonas* ↑ *Halomonas*	16S rRNA gene analysis and next-generation sequencing	[65]
Human	Extrahepatic cholangiocarcinoma	Bile microbiota Biliary fluid	↑ *Bacteroides* ↑ *Geobacillus* ↑ *Meiothermus* ↑ *Anoxybacillus*	16S rRNA sequencing	[66]
Human	Distal cholangiocarcinoma	Bile microbiota Bile sample	↑ *Gemmatimonas* ↑ *Nitrospira* ↑ *Chloroflexus* ↑ *Planctomyces*	16S rRNA sequencing	[67]
Human	Cholangiocarcinoma	Bile microbiota Bile duct tissue Gastric microbiota Gastric mucosal tissue	↑ *Dietziaceae* ↑ *Pseudomonadaceae* ↑ *Oxalobacteraceae* ↑ *Moraxellaceae* ↓ *Burkholderiaceae*	16S rRNA sequencing	[68]

NAFLD, non-alcoholic fatty liver disease; NASH, non-alcoholic steatohepatitis; ↑ Increased bacterial abundance; ↓ Decreased bacterial abundance.

## Data Availability

No new data were created or analyzed in this study. Data sharing is not applicable to this article.
